# Prolongation of life by adoptive cell therapy with cascade primed immune cells in four patients with non-small cell lung cancer stages IIIB and IV and a pancoast tumor: a case series

**DOI:** 10.1186/1752-1947-7-266

**Published:** 2013-12-11

**Authors:** Barbara Laumbacher, Songhai Gu, Rudolf Wank

**Affiliations:** 1 Immunotherapy Research Center, Pettenkoferstrasse 8, 80336 München, Germany

## Abstract

**Introduction:**

Despite newer treatment modalities, few patients with non-small cell lung cancer in stages IIIB and IV survive the median of one year. We present four patients with non-small cell lung cancer treated with an adjuvant therapy with cascade primed immune cells. The *in vitro* stimulated expression of cancer information on the patients’ monocytes matures and activates T lymphocytes to destroy cancer cells. The cascade primed immune cell therapy significantly improved the quality of life and the lifespan of all four patients; thus far, three patients survived 40, 55 and 120 months, respectively; and one patient died 39 months after diagnosis.

**Case presentation:**

Patient 1, stage IV (T4N2M1): The adenocarcinoma of the 67-year-old German Caucasian man infiltrated into the mediastinal lymph nodes and iliosacral bones. Chemotherapy modalities were started immediately after diagnosis of cancer, and cascade primed immune cell therapy one year later. The patient survived 39 months.

Patient 2, stage IV (T3N3M1a): The 62-year-old German Caucasian woman presented with adenocarcinoma of the lower lobe with infiltrated lymph nodes of the mediastinum and malignant pleural effusion. Chemotherapy, radiation and the cascade primed immune cell therapy were administered together. The patient is still alive after 40 months.

Patient 3, stage IIIB (T4N1-2M0): The 75-year-old German Caucasian woman presented with an undifferentiated tumor and a separate tumor nodule in the ipsilateral lobe. The patient received only cascade primed immune cell therapy after tumor resection and has survived for the last 55 months.

Patient 4, pancoast tumor (IIIB, T3N3M0): The 77-year-old German Caucasian man presented with an undifferentiated tumor that infiltrated the lymph nodes, the clavicle, one rib and the plexus brachialis. In addition to chemotherapy and radiation, cascade primed immune cells were administered every weekday for one year. After four months, no living tumor cell was detected in the resected lung, the lymph nodes or the bone material. The patient is still alive after 120 months.

**Conclusions:**

The novel adoptive cell therapy with cascade primed immune cells significantly increased the survival rate and maintained the quality of life for four patients with non-small cell lung cancer in stages IIIB and IV. Our findings indicate that tumor resection, chemotherapy and radiation appear to support the cascade primed immune cell therapy.

## Introduction

The interest in adoptive cell therapy (ACT) is continuously growing for three reasons. First, Rosenberg *et al.* reported convincing clinical results of ACT in patients with tumor-infiltrating lymphocytes (TIL) who were treated for melanoma [1]; and Takayama *et al*. reported their findings for the treatment of liver cancer [2]. Second, in solid cancers, particularly in non-small cell lung cancer (NSCLC), chemotherapy and radiation as well as newer modalities achieve only a modest prolongation of survival time. Third, this modest prolongation of survival is accompanied by severe side effects that often heavily affect the quality of the patient’s shortened life. In ACT, two strategies are most often used to stimulate and armour autologous immune cells against cancer cells. One strategy is the *in vitro* activation of T lymphocytes, which is based on the fact that the T cells in malignant tumors recognize the cancer cells but they do not destroy the cancer. As described by Rosenberg *et al*., such TIL have been isolated, resuscitated and amplified *in vitro* and subsequently infused into the patients [1]. Another strategy employs the patient’s dendritic cells, which are prepared *in vitro* with different methods to express cancer peptides that can activate the naïve/resting T lymphocytes *in vivo*[3]. The cascade primed immune (CAPRI) cell method uses elements of both strategies.

Antigen-presenting cells (APC) internalize, process and present foreign and autologous proteins; however, until recently, it was not considered that APC could supply information about autologous cancer proteins. If T lymphocytes were activated with OKT3 antibodies in peripheral blood mononuclear cell (PBMC) bulk cultures, they stimulate the processing and the presentation of tumor-immunogenic information. These stimulated monocytes could mature autologous naïve/resting lymphocytes to cytotoxic effector cells. In another published study, we demonstrated that activated monocytes differentiated naïve/resting T lymphocytes to potent cancer-destructing effector T cells *in vitro*, which significantly prolonged the lifespan of patients with breast cancer [4]. In this study, we describe the successful life prolongation of four patients with NSCLC in stages IIIB and IV, who were treated with the CAPRI cells in combination with surgical intervention and/or chemotherapy and radiation.

All steps of the cell preparations, including the final therapy (the treatment attempts), were controlled by a medical doctor (RW). In Germany, treatment attempts with new modalities are permitted if they are performed on the authority of a medical doctor [4]. An institutional review was not required. Patients were informed about the experimental nature of the approach, and the ethics recommendations of Helsinki along with subsequent amendments of Tokyo 1975, Hong Kong 1989 and Somerset West 1996 were followed.

## Case presentation

### Patient 1

#### Oncological diagnosis

The 67-year-old German Caucasian man presented with a tumor of the upper lung lobe that infiltrated the mediastinum and the mediastinal lymph nodes, and with metastases of the iliosacral bones. Evaluations of the tumor size (T4), regional lymph node involvement (N2) and distant metastases (M1) (T4N2M1) led to a diagnosis of NSCLC in stage IV. A histological analysis revealed adenocarcinoma. Locoregional progress was seen 10 months after diagnosis (AD), malignant pleural effusion 12 months AD, locoregional progress and suspicion of brain metastases 20 months AD, tumor infiltration of the thorax walls and malignant pleural effusion 32 months AD.

#### Therapy

**Figure 1:**
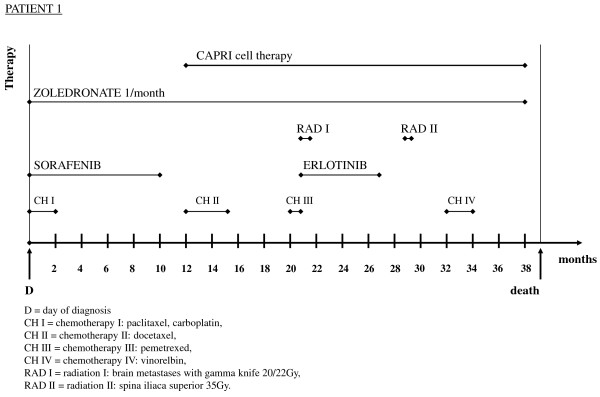
**Survival time and therapeutic modalities of four patients with non-small cell lung cancer.** Shown are months of survival after diagnosis and therapeutic modalities in chronological order. CAPRI, cascade primed immune.

Therapy was started immediately with injections of the biphosphonate zoledronate and a combined chemotherapy with paclitaxel, carboplatin and sorafenib (Figure 1). The combined modality was stopped two months AD because of diarrhea, vomiting, progressive weakness and tumor growth. Sorafenib was continued and stopped 10 months AD because the disease progressed. Docetaxel was started 12 months AD and stopped 15 months AD because of paravasate application and leg edema despite a marginal tumor reduction. Pemetrexed was started 20 months AD and stopped after one round because of the same side effects. Two brain metastases were irradiated with the gamma knife (20Gy/22Gy) two weeks later. Treatment with the tyrosine kinase inhibitor erlotinib was started 21 months AD and stopped after six months because of leg edema, nose bleeding and peripheral neuropathies. The latter condition was treated with gabapentin unsuccessfully. A malignant pleural effusion was drained 25 months AD; irradiation of the right spina iliaca superior (35Gy) and an infusion of two red cell concentrates were conducted because of chemotherapy-associated anemia 29 months AD. A therapy with vinorelbine was started 32 months AD and continued until 34 months AD (Figure 1).

ACT with the CAPRI cells started 12 months AD. Three months after the CAPRI cell therapy, a marginal reduction of the tumor was noticed in the computed tomography (CT) scan. The patient survived 39 months; over a period of 26 months he received, twice weekly, 268 injections comprising 60 to 80 million CAPRI cells, two-thirds intravenously, one-third intracutaneously. The cell donations were occasionally interrupted for a week during the patient’s hospitalization. The CAPRI cell therapy was hampered not only by the low leucocyte count but also by the low quality of the immune cells. The patient survived 39 months.

### Patient 2

#### Oncological diagnosis

The 62-year-old German Caucasian woman presented with a nonresectable adenocarcinoma of the right lower lobe with infiltrated lymph nodes of the mediastinum and malignant pleural effusion, staged by tumor size and dissemination (T3N3M1a) as IV.

The first CT scan 12 months AD showed a stable disease; a positron emission tomography/computed tomography (PET/CT) scan 20 months AD exhibited a marginal but definite progression of the disease, disintegration of the central lymph nodes without enlargement, pleural effusion but no distant metastases. A PET/CT scan 26 months AD, performed because of swallowing difficulties, revealed an enlarged lymph node that had caused a severe obstruction of the esophagus, which required treatment with a stent. A histological analysis revealed tumor infiltration, which had caused the lymph node enlargement. Neither a second radiation nor chemotherapy was performed.

#### Therapy

The patient received a combined treatment of radiation and chemotherapy for three months starting two weeks AD. Four cycles of cisplatin and pemetrexed were administered together with a total of 64Gy.

**Figure 2:**
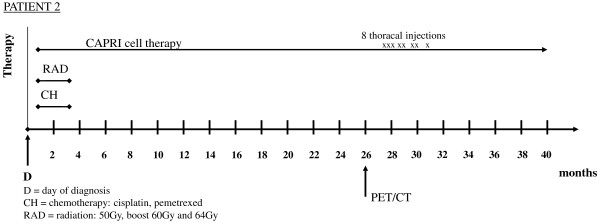
**Survival time and therapeutic modalities of a 62-year-old German Caucasian woman with non-small cell lung cancer.** Shown are months of survival after diagnosis and therapeutic modalities in chronological order. CAPRI, cascade primed immune; PET/CT, positron emission tomography/computed tomography.

ACT with the CAPRI cells started after radiation and chemotherapy, that is, three months AD (Figure 2). In the first six months, the patient received 40 to 60 million CAPRI cells twice weekly; after improvement of the cell count, the patient received 80 million CAPRI cells twice weekly, two-thirds intravenously, one-third intracutaneously, with a total of 260 injections over a period of 35 months. The growing pleural effusion, diagnosed by PET/CT scan 26 months AD, was first treated for several months with thoracentesis and later successfully combined with thoracic CAPRI cell injections eight times using 100 to 300 million immune cells. After the first thoracic injection of 300 million CAPRI cells, the patient felt very weak and had a low-grade fever for two days. Consequently, the dose of CAPRI cells was reduced to 100 million, which was well tolerated. The patient is still alive after 40 months (Figure 2).

### Patient 3

#### Oncological diagnosis

The 75-year-old German Caucasian woman presented with an undifferentiated tumor ‘resembling’ a squamous cell carcinoma of the upper lobe and a separate tumor nodule in the ipsilateral tumor lobe (T4N1-2M0, stage IIIB). The PET/CT scan restaging each year had shown no suspicious growth of the tumor until now.

#### Therapy

The patient refused chemotherapy or radiation but agreed to surgery. The upper lobe was resected two months AD, the middle lobe was resected six months AD because of an aspergilloma.

ACT with CAPRI cells was begun after the tumor resection (two months AD); the patient received 152 injections of the CAPRI cells; during the first year, she received 60 to 80 million CAPRI cells twice weekly, subsequently once a week.

**Figure 3:**
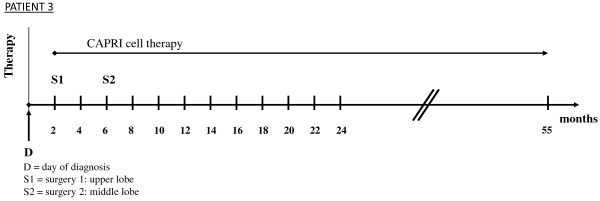
**Survival time and therapeutic modalities of a 75-year-old German Caucasian woman with non-small cell lung cancer.** Shown are months of survival after diagnosis and therapeutic modalities in chronological order. CAPRI, cascade primed immune.

The patient is in good condition and has survived for the last 55 months (Figure 3).

### Patient 4

#### Oncological diagnosis

The 77-year-old German Caucasian man presented with a large cell undifferentiated pancoast tumor (IIIB, T3N3M0) of the left upper lobe that infiltrated the lymph nodes, the clavicle, the first rib and the plexus brachialis; no distant metastasis was found. A PET/CT scan was performed every three months in the first year AD, in the second year every six months and subsequently each year. No ^18^F-FDG uptake was seen in the examinations after surgery. Before the PET/CT scan, the CAPRI cell therapy had to be interrupted for two weeks to avoid unspecific uptake of ^18^F-FDG by CAPRI cells in the tumor region.

#### Therapy

Over the course of five months, the patient received the maximum radiation dose of 70Gy in combination with 25 rounds of cisplatin. In the subsequent surgery six months AD, the upper lobe, the first rib, the clavicle and a portion of the pleura were resected, but it was not possible to remove the portions of the tumor that enveloped the plexus brachialis.

**Figure 4:**
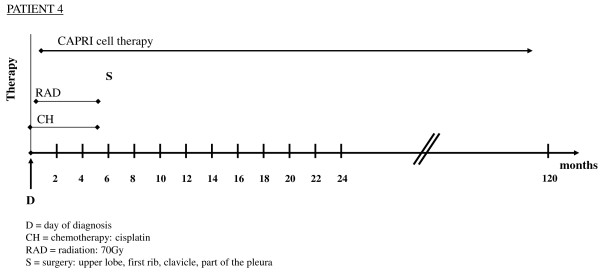
**Survival time and therapeutic modalities of a 77-year-old German Caucasian man with non-small cell lung cancer.** Shown are months of survival after diagnosis and therapeutic modalities in chronological order. CAPRI, cascade primed immune.

ACT with 100 to 120 million CAPRI cells was administered every weekday until surgery, one third intracutaneously, two-thirds intravenously. Because the tumor could not be completely removed, the CAPRI cell therapy was continued for an entire year with the same frequency and intensity. No negative side effects were observed despite the large amounts of activated immune cells. The patient received, in the first year, 223 injections of CAPRI cells; he still receives 20 to 40 million of CAPRI cells one to two times per week, with interruptions for excursions and other endeavors. For several years after the surgery, the patient has enjoyed playing golf and is still alive after 120 months (Figure 4).

## Discussion

The therapy of patients with NSCLC in stages IIIB and IV remains unsatisfactory, despite improvements with combined radiation and chemotherapy and newer modalities. We report the successful adjuvant treatment with the CAPRI cells for four patients in stages IIIB and IV. All four patients surpassed the median survival time of nine to 13 months [5,6]. The patient in stage IV died 39 months AD; the other patients have survived up to the time of this publication 40, 55, and 120 months, respectively. The patients reported that they were able to resume their normal activities, such as long walks, bicycling or playing golf after they had received the CAPRI cell injections (patients 2, 3 and 4). An adverse effect was observed in patient 2 after the thoracic injection of 300 million CAPRI cells. She responded with fever and tiredness for two days. The thoracic administration of lower cell numbers, that is, 100 million CAPRI cells, was well tolerated.

The preparation of CAPRI cells differs from other ACT methods and has been previously described [4]. The immune cells are isolated from the peripheral blood. The T lymphocytes are activated *in vitro* with the monoclonal antibody CD3, in the presence of other immune cells, especially monocytes. The activated T lymphocytes stimulate monocytes to present the phagocytozed tumor material. It is very important to add ‘new’ unstimulated T lymphocytes to the stimulated monocytes because CD3-activated T lymphocytes internalize the antigen-T-cell receptor and cannot recognize the cancer-relevant information of monocytes. Using proper timing in the activation steps yields the CAPRI cells after 24 hours. These CAPRI cells recognize and destroy cancer cells and are ready to be injected into patients. However, in the majority of the cases, larger numbers of CAPRI cells are needed; the CAPRI cells are cultured for one week. The addition of the cytokine interleukin-2 induces cell division and an increase in the number of CAPRI cells. The CAPRI cells can be produced for cancer patients without loss of time because no tumor-immunogenic peptides need to be identified, which must be inoculated into APC. Furthermore, the T lymphocytes can be used from the peripheral blood and can be easily expanded. The T lymphocytes from the blood are not damaged by the tumor environment as in the case of TIL. Finally, we have tested many different types of cancer and found that the monocytes of other cancers also present tumor-immunogenic determinants [4].

The point of time for isolating the immune cells for the preparation of the CAPRI cells influences the efficiency of the CAPRI cells. For the success of ACT, it is important to isolate the immune cells before they are damaged by radiation and chemotherapy. In patients 1 and 2, the immune cells were isolated after completion of the first rounds of chemotherapy and radiation; that is, the immune cells and/or their precursors in the bone marrow were exposed to chemotherapeutical reagents and/or radiation therapy. Another influential factor is the presence of a large tumor mass; in patients 1 and 2, the tumor could not be removed or reduced by surgery. A larger tumor mass had to be attacked by the immune cells. Understandably, it is not possible to derive strong conclusions from four patients. We do not know whether patient 1 would have survived longer if the immune cells were isolated from his blood before chemotherapy. Although patient 1 was the only one with distant metastasis, it is worthy of note that the median survival of NSCLC patients with stage IV seems to differ only minimally from patients with stage IIIB [5,6]. In patient 2 (stage IIIB), chemotherapy and radiation preceded the immune cell isolation. The tumor could not be resected. Although the patient shows a prolonged survival time of 40 months with a stabilization of the disease, including a marginal tumor reduction, a local regrowth of the tumor was recognized in the PET/CT scan in the 36th month. In contrast, patient 3 (stage IIIB) and patient 4 (pancoast tumor) are still in complete remission 55 and 120 months AD without signs of recurrence. Patient 3 had decided to undergo only surgery, in addition to the CAPRI cell therapy, whereas patient 4 had a combined chemotherapy and radiation but his CAPRI cells were prepared from immune cells, which had been isolated before the start of chemotherapy and radiation. With all of the caveats of immunogenetic variations among these patients, variations of tumor size, tumor type and the total numbers of CAPRI cells injected in these four patients, the disease course in patients 3 and 4 suggests that there is an advantage in isolating immune cells before chemotherapy and radiation. Furthermore, the resection and reduction of the tumor, if possible, seem to be advantageous in stages IIIB and IV.

Another aspect appears in patient 4. In the resected tumor material, no living cancer cell was found after six months, neither in the tumor itself nor in the clavicle, rib or lymph nodes. This observation suggests a synergism of chemotherapy and radiation with a simultaneous CAPRI cell therapy. The halt of tumor growth by chemotherapy or radiation may support the downsizing of the tumor by the CAPRI cells. Furthermore, the inflammation caused by irradiation and chemotherapy may enhance migration to the tumor and the cytotoxic activity.

## Conclusions

Cancer cells of the NSCLC type appear to be excellent immunogenic targets for the CAPRI cells. The inflammation caused by chemotherapy and radiation may be an additional stimulus for cytotoxic attacks by the CAPRI cells. The reduction of the cancer mass by surgery or the arrest of tumor growth by chemotherapy and radiation seems to support the tumor downsizing by the CAPRI cells. For the achievement of a synergism between the CAPRI cells and other modalities, it is important to isolate the immune cells before chemotherapy, radiation or other treatment modalities, which might damage the bone marrow and the immune cells.

## Consent

Written informed consent was obtained from each of the three surviving patients and from the wife of the deceased patient for publication of this case series and any accompanying images. A copy of the written consents are available for review by the Editor-in-Chief of this journal.

## Ethical Approval

IRB approval was not required as, in Germany, treatment attempts with new modalities are permitted, if they are performed on the authority of the medical doctor [4]. The authors hereby confirm that the treatment was performed solely to meet the patients’ clinical needs, at the discretion of the responsible physician (RW). Confirmation of this is available for the Editor in Chief of the journal to check.

## Competing interests

RW holds the European and international patents on the CAPRI procedure.

## Authors’ contributions

BL, SG and RW were involved in acquiring data and writing the manuscript; RW designed the concept of the treatment. All authors read and approved the final manuscript.
